# Mechanisms and Clinical Implications of Endothelial Dysfunction in Arterial Hypertension

**DOI:** 10.3390/jcdd9050136

**Published:** 2022-04-27

**Authors:** Pasquale Ambrosino, Tiziana Bachetti, Silvestro Ennio D’Anna, Brurya Galloway, Andrea Bianco, Vito D’Agnano, Antimo Papa, Andrea Motta, Fabio Perrotta, Mauro Maniscalco

**Affiliations:** 1 Istituti Clinici Scientifici Maugeri IRCCS, Cardiac Rehabilitation Unit of Telese Terme Institute, 82037 Telese Terme, Italy; antimo.papa@icsmaugeri.it; 2 Istituti Clinici Scientifici Maugeri IRCCS, Scientific Direction, 27100 Pavia, Italy; tiziana.bachetti@icsmaugeri.it; 3 Istituti Clinici Scientifici Maugeri IRCCS, Pulmonary Rehabilitation Unit of Telese Terme Institute, 82037 Telese Terme, Italy; silvestro.danna@icsmaugeri.it; 4 Department of Translational Medical Sciences, University of Campania “Luigi Vanvitelli”, 80131 Naples, Italy; brurya29@gmail.com (B.G.); andrea.bianco@unicampania.it (A.B.); vito.dagnano94@gmail.com (V.D.); fabio.perrotta@unicampania.it (F.P.); 5 Institute of Biomolecular Chemistry, National Research Council, 80078 Pozzuoli, Italy; andrea.motta@icb.cnr.it; 6 Department of Clinical Medicine and Surgery, “Federico II” University, 80131 Naples, Italy

**Keywords:** arterial hypertension, endothelial dysfunction, occupational medicine, heart failure, chronic disease, arginine, rehabilitation, exercise, outcome, cardiovascular disease

## Abstract

The endothelium is composed of a monolayer of endothelial cells, lining the interior surface of blood and lymphatic vessels. Endothelial cells display important homeostatic functions, since they are able to respond to humoral and hemodynamic stimuli. Thus, endothelial dysfunction has been proposed as a key and early pathogenic mechanism in many clinical conditions. Given the relevant repercussions on cardiovascular risk, the complex interplay between endothelial dysfunction and systemic arterial hypertension has been a matter of study in recent years. Numerous articles have been published on this issue, all of which contribute to providing an interesting insight into the molecular mechanisms of endothelial dysfunction in arterial hypertension and its role as a biomarker of inflammation, oxidative stress, and vascular disease. The prognostic and therapeutic implications of endothelial dysfunction have also been analyzed in this clinical setting, with interesting new findings and potential applications in clinical practice and future research. The aim of this review is to summarize the pathophysiology of the relationship between endothelial dysfunction and systemic arterial hypertension, with a focus on the personalized pharmacological and rehabilitation strategies targeting endothelial dysfunction while treating hypertension and cardiovascular comorbidities.

## 1. Introduction

Systemic arterial hypertension (SAH) is the substrate of many cardiovascular and systemic disorders, leading to structural or functional impairment of the arterial vasculature and/or the organs it supplies [1]. Main end organ damage due to uncontrolled hypertension may affect the brain, heart, kidneys, central and peripheral arteries, and the eyes [2]. SAH has emerged as a major health problem because of the progressive growth in the ageing population coupled with the increased prevalence of predisposing risk factors, such as obesity, salt consumption, physical deconditioning, and inactivity [3]. The pathophysiology of hypertension is particularly complex and multifactorial and may be associated in a bidirectional relationship with endothelial dysfunction [4].

The endothelium is a thin layer of flat polygonal cells strategically located between the bloodstream and the vascular smooth muscle wall, exerting essential functions for the maintenance of vascular homeostasis [5]. Endothelial dysfunction is a phenotypic modification in the endothelium, leading to exalted prothrombotic and proinflammatory status [6]. The interaction between SAH and endothelial dysfunction may act at different levels: firstly, the compromised endothelial cells promote an altered reactivity of the vascular smooth muscle tone; secondly, the prothrombotic and proinflammatory phenotype induced by endothelial cell dysfunction amplifies the systemic effects of arterial hypertension, thus leading to end organ damage [7]. Currently, it is commonly believed that the relationship between endothelial dysfunction and hypertension is not linear, as each factor could influence the other, giving rise to a pathogenetic vicious circle [4]. Original research has documented that the integrity of endothelial cell function may control vascular smooth muscle tone in response to various agents, including acetylcholine, calcium ionophore, adenosine triphosphate (ATP), adenosine diphosphate (ADP), substance P, bradykinin, histamine, and thrombin [8]. Additionally, endothelial cells dynamically secern a plethora of mediators, including endothelins, cyclooxygenase-dependent vasoconstrictors, and endothelium-derived hyperpolarizing factors, that are involved in the pathophysiology of hypertension [5]. However, the prognostic significance of assessing endothelial dysfunction in hypertension is yet to be established [4].

In this review, we aimed to describe the molecular mechanisms involved in this complex interplay and define possible therapeutic targets modulating endothelial dysfunction in hypertensive patients.

## 2. Endothelial Cell Mediators in the Pathogenesis of Arterial Hypertension

A healthy endothelium releases a variety of factors in order to guarantee the appropriate vascular tone, the maintenance of a non-adhesive and unabridged surface, to prevent vascular remodeling, and to regulate the formation of new vessels [5]. Particularly, the vascular tone is balanced by endothelial-derived vasodilators and vasoconstrictors [9].

### 2.1. Endothelial-Derived Vasoactive Mediators

Among the vasodilator factors, a key player is undoubtedly nitric oxide (NO). NO is synthesized by the endothelial enzyme nitric oxide synthase (eNOS) starting from L-arginine and oxygen in the presence of several cofactors [6]. NO is a gas, freely diffusible and highly reactive. It induces vasodilation in the underlying smooth muscle cells by interacting with soluble guanylate cyclase, which activates a cascade of molecular pathways that ultimately lead to reduced intracellular calcium and increased intracellular potassium, favoring cell membrane hyperpolarization and muscle relaxation [10]. NO also has an antiproliferative effect on vascular smooth muscle cells [11]. When NO diffuses to the luminal side of the endothelial monolayer, it exerts an antithrombotic action by inhibiting platelet adhesion and aggregation [10]. Moreover, NO also prevents leukocyte adhesion to vascular endothelium and leukocyte migration into the vascular wall, thus exerting a physiological anti-atherosclerotic action [12].

Endothelial dysfunction is characterized by reduced release or availability of NO, which results in impaired endothelium-dependent vascular relaxation [10]. Therefore, endothelial dysfunction has been largely documented in hypertension [13,14]. The Framingham study was one of the first population-based studies showing that systolic blood pressure was inversely correlated with flow-mediated dilation (FMD) [15], which is largely accepted as an accurate, cost-effective, and noninvasive method to assess endothelial function in humans [16]. Although the study design could not allow the determination of a cause–effect relationship, the Framingham study demonstrated the presence of a link between these two conditions. Several factors may affect the production and bioavailability of NO. Oxidative stress can cause eNOS uncoupling due to reduced availability of the enzyme cofactor tetrahydrobiopterin (BH4) and deficiency of the substrate L-arginine, with the consequent production of superoxide radicals instead of NO [17]. Superoxide radicals scavenge NO, producing the toxic radical peroxynitrite. Thus, reactive oxygen species (ROS), which include also peroxides and hydroxyl radicals, have an important role in the homeostasis of vascular wall and they are likely factors promoting hypertension [18]. ROS are mainly produced in the cardiovascular and renal systems by a family of nicotinamide adenine dinucleotide phosphate (NADPH) oxidases (NOX) [19,20]. Several NOX isoforms have been shown to be involved in progression of hypertension in animal models [20]. Additionally, endoplasmic reticulum stress and mitochondrial oxidative stress also contribute to endothelial dysfunction and vascular remodeling in hypertension [21,22]. Although a causative link between ROS and increased blood pressure has not been demonstrated in hypertensive patients, positive associations between systemic biomarkers of oxidative stress and blood pressure values have been observed, as well as a reduced antioxidant capacity [23,24].

In addition to NO, the endothelium-derived hyperpolarizing factor (EDHF) can induce vascular relaxation [25]. Its chemical nature is unknown but is presumed to be either a chemical mediator or an electrical transducer, depending on the species and vascular beds considered. EDHF induces opening of Ca^2+^-activated K^+^ channels, thus hyperpolarizing the membrane potential of vascular smooth muscle cells, especially in resistance microvessels [26]. EDHF’s potential role in the development of either human or animal hypertension is currently unknown.

Among endothelium-derived vasoconstrictors, the peptide endothelin-1 (ET-1) and angiotensin converting enzyme (ACE) play key roles. Endothelial cells generate ET-1 in response to several stimuli, such as ROS, inflammatory molecules, and hypoxia [27]. ET-1 can prompt vascular constriction by activating ET_A_ and ET_B2_ receptors on smooth muscle cells [28]. The vasoconstriction effect is mediated by increased intracellular calcium concentration and phosphorylation, resulting in myosin light chain activation [29]. ET-1 is also a potent mitogen able to stimulate the growth, proliferation, and migration of smooth muscle cells, with important implications in vascular remodeling [30]. However, ET-1 also has counter-regulatory properties, as it is able to interact with ET_B1_ receptors on the endothelial membrane and activate a signaling cascade, resulting in NO and prostacyclin (PGI_2_) production, with consequent vascular relaxation [31]. ET-1 is constantly released by the endothelium but its concentrations are important in determining vascular function. In fact, low levels of ET-1 promote vasodilation, while high levels of ET-1 increase blood pressure and peripheral vascular resistance [32,33]. When the endothelium is dysfunctional, the balance between ET-1 vasodilator/vasoconstrictor effects is disrupted in favor of the latter [34]. Both experimental and clinical studies have shown high levels of ET-1 in hypertension, suggesting the presence of a link between ET-1 levels and development of systemic hypertension [35]. In addition, ET-1 is directly involved in the process of arterial remodeling causing hypertrophic thickening of small arteries [36]. The increased vascular wall thickness combined with the increased tone bring about increased peripheral vascular resistance, a typical hallmark of hypertension [37]. Moreover, ET-1 is also able to induce vascular inflammation, stimulating the expression of NOX in vascular cells with subsequent increased production of ROS, which, in turn, promotes the synthesis and release of inflammatory molecules, such as cytokines and adhesion molecules [38]. The inflammatory process is further amplified by the recruitment and activation of circulating immune cells by ET-1 [28].

**Figure 1:**
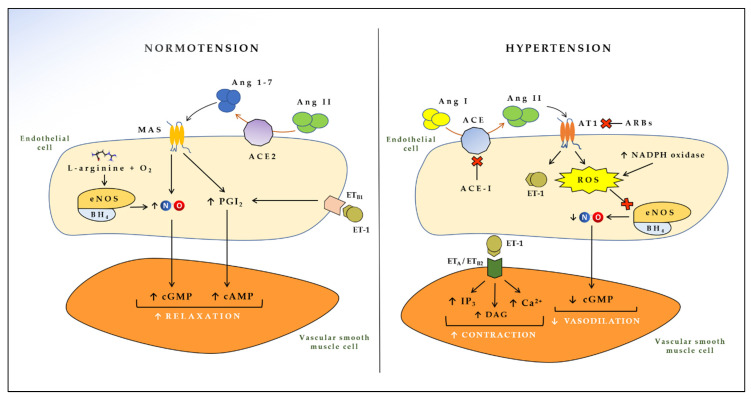
Effects of endothelial-derived vasoactive mediators in the cross-talk between endothelial and vascular smooth muscle cells. ACE: Angiotensin converting enzyme; ACE2: Angiotensin converting enzyme 2; Ang I: Angiotensin I; Ang II: Angiotensin II; Ang 1-7: Angiotensin 1-7; AT1: Angiotensin II type 1 receptor; MAS: Proto-oncogene G-protein-coupled receptor; eNOS: Endothelial nitric oxide synthase; BH4: Tetrahydrobiopterin; ET-1: Endothelin 1; ET_B1_: Endothelin receptor B1; ET_B2_: Endothelin receptor B2; ET_A_: Endothelin receptor A; NO: Nitric oxide; PGI_2_: Prostacyclin; NADPH: Nicotinamide adenine dinucleotide phosphate; ROS: Reactive oxygen species; cGMP: Cyclic guanosine monophosphate; cAMP: Cyclic adenosine monophosphate; IP_3_: Inositol triphosphate; DAG: Diacylglycerol; ACE-I: Angiotensin-converting enzyme inhibitors; ARBs: Angiotensin II receptor blockers.

ACE is constitutively expressed by the vascular endothelium and is particularly abundant in the lungs [39]. ACE is of paramount importance in the development of hypertension, since ACE is able to convert angiotensin I into angiotensin II (Ang II), a polypeptide with several biological effects in vascular smooth muscle cells [40]. Ang II interacts with the Ang II type I (AT1) receptor and activates a cascade of intracellular pathways that results in increased production of ROS, release of growth factors, release of ET-1 and adhesion molecules, triggering endothelial impairment, vasoconstriction, and remodeling of resistance arteries [41,42], ultimately leading to hypertension [43]. A physiological counter-regulatory pathway is activated when Ang II interacts with Ang II type II (AT2) receptor [44]. In this case, the biological effects elicited are opposite to the ones just described, eventuating in vasodilation and other homeostatic effects [45]. However, the affinity of Ang II for AT2 receptor is lower than that for AT1 receptor, making the first pathway prevalent over the second one, with consequent detrimental effects on blood pressure [45]. Another escape pathway for Ang II, which has recently come into prominence due to severe acute respiratory syndrome coronavirus-2 (SARS-CoV-2), is given by ACE2, an enzyme (and receptor for the coronavirus spike protein) able to transform Ang II into angiotensin 1-7 [46,47]. This very short peptide interacts with the proto-oncogene G-protein-coupled MAS receptor on endothelial cells, leading to higher NO availability and reduced ROS, with consequent beneficial effects on blood pressure [13,48] (Figure 1).

### 2.2. Extracellular Vesicles in the Cross-Talk between Endothelial and Smooth Muscle Cells

Besides traditional molecular effectors, other players able to modulate endothelial and vascular function have come to the arena in the last years. Among these, are the extracellular vesicles (EV).

EV are particles naturally released from various cell types that are unable to replicate [26]. They may contain a heterogeneous cargo of material ranging from microRNAs, long noncoding RNAs, DNA fragments, transcription factors, ROS, proteins, metabolites, and lipids [49]. EV can be divided into three subcategories: exosomes, microvesicles, and apoptotic bodies [50,51]. Exosomes are small EV (diameter: 40–160 nm), while microvesicles are large EV (diameter: 0.1–1 µm). Only the microvesicles are encased by a characteristic plasma membrane, while exosomes are delimited by endosomal membranes and are directly released by the cells to the extracellular space [50]. Apoptotic bodies are composed of discard material, such as intracellular fragments and damaged organelles, enveloped by plasma membranes [52]. Following binding to cells, circulating exosomes and microvesicles fuse with extracellular plasma membranes or internalize and release their content to the recipient cells [51].

The endothelium is both a recipient and a generator of EV [53], exerting multiple effects in the progression of hypertension, ranging from reduced NO release and increased ROS production to stimulation of proliferation and migration of vascular smooth muscle cells [54]. EV may act in a paracrine or endocrine fashion and affect endothelial function at sites distant from their production, thus potentially representing a promising biomarker in endothelial dysfunction assessment [54]. Experimental studies have shown that infusion of concentrated EV is able to impair vasodilation in resistance arteries of normotensive animals [55] and that infusion of exosomes from spontaneously hypertensive rats increased systolic pressure of normotensive animals [56]. Results from clinical studies are in line with animal studies and show high circulating levels of endothelial- and platelet-derived EV in hypertensive patients [57,58], suggesting the involvement of EV in the pathogenesis of hypertension [54].

EV levels undoubtedly directly correlate with systolic blood pressure, arterial diameter, and pulse wave velocity [57]. In addition to EV levels, it is also important to take into account the composition of EV. A recent study showed in hypertensive patients with albuminuria that the profile of 29 plasma exosomal microRNAs is different from that of control subjects [59]. Animal studies have further provided mechanistic insights into the arterial remodeling induced by differently expressed microRNAs in EV of hypertensive versus normotensive animals [60].

## 3. Endothelial Dysfunction, Hypertension, and Cardiovascular Risk

The close inter-relationship between endothelial dysfunction and hypertension may represent the main pathogenic mechanism of small vessel disease in vital organs (e.g., heart, brain, kidney) [13]. Animal models of hypertension and cell culture studies have shown that, although endothelial and microvascular dysfunction are not exactly the same, an injured endothelium represents the earliest stage of an impaired functioning of the other vascular components (e.g., smooth muscle cells) [61]. The current tendency is to interpret small vessel disease as a systemic disorder with a common pathogenic background that differentially affects isolated organs [62]. Thus, cerebral small vessel disease is seen as the leading cause of cognitive decline and ischemic complications, being frequently observed also in Alzheimer’s disease [61]. Similarly, hypertensive coronary microvascular dysfunction has been identified as a subclinical marker of end organ damage and heart failure [63]. The evidence that peripheral microvascular endothelial dysfunction is associated to cerebral small vessel disease, thus potentially predicting the risk of future stroke [64], supports the hypothesis that endothelial dysfunction reflects a systemic process of vascular remodeling initiated by hypertension and other cardiovascular risk factors [65]. This is further confirmed by the observation that peripheral endothelial dysfunction is able to predict the severity of cerebral small vessel disease even when evaluated in conduit arteries [66]. Conversely, the fact that microvascular dysfunction may be able to affect blood pressure and flow patterns is in line with the less traditional hypothesis that endothelial damage and subsequent microvascular dysfunction are causes rather than consequences of hypertension [67].

Overall, it is evident that peripheral endothelial function and arterial pressure are responsible for blood supply to the periphery and, therefore, for protection from cardiovascular events [68]. In a landmark study [69], endothelium-dependent and endothelium-independent coronary vasoreactivity were tested through intracoronary instillation of acetylcholine in 147 patients with a median follow-up period of 7.7 years. The authors demonstrated that the incidence of cardiovascular events (cardiovascular death, unstable angina, myocardial infarction, coronary revascularization, ischemic stroke, and peripheral artery revascularization) was lower in subjects with preserved endothelial responsiveness. Therefore, endothelial dysfunction emerged as a phenotype with high risk for cardiovascular events. In keeping with this, Yeboah et al. [70] examined 3026 subjects without cardiovascular disease from the Multi-Ethnic Study of Atherosclerosis (MESA) cohort, showing that each standard deviation increase in FMD corresponded to a hazard ratio of 0.84 for incident cardiovascular events after 5 years. These data were corroborated from another clinical research from Gokce et al. [71] in patients undergoing peripheral or coronary bypass surgery. Authors demonstrated a higher risk of postoperative cardiac events in subjects with impaired endothelial function, expressed by a low FMD (i.e., FMD < 8.1%). Using the MESA cohort, Shimbo et al. [72] were among the first demonstrating an association between FMD and hypertension. Moreover, when specifically considering participants without hypertension, the authors also documented a significant association between baseline FMD and incident hypertension at 4.8-year follow-up. However, the latter finding was not confirmed in multivariate analyses. In another study [73], the coexistence of left ventricular hypertrophy and endothelial dysfunction in hypertensive patients emerged as risk for subsequent major cardiovascular events. In particular, hypertensives with left ventricular hypertrophy showed attenuated brachial and coronary artery endothelium-dependent vasodilation, suggesting that both the endothelium and left ventricle may be damaged by hypertension. This study supports the hypothesis that hypertension may have a causal role in endothelial dysfunction, which is the earliest stage of atherosclerosis, thereby representing one of the main traditional cardiovascular risk factors [74]. This is in line with the evidence that, when NO synthase antagonists are administered to normotensive subjects, a significant increase in systemic blood pressure can be documented [75]. Accordingly, the Cardiovascular Risk in Young Finns Study found that hypertension in youth may predict future impaired endothelial function [76]. The widely accepted viewpoint that hypertension is a cause rather than a consequence of endothelial dysfunction is in contrast with the evidence on 957 postmenopausal women in which the incidence of hypertension at 3.6-year follow-up was nearly sixfold higher in those in the lowest FMD quartile, with a 16% increase in cardiovascular risk per unit of FMD [77]. The relatively healthy cohort of postmenopausal women is a strength of this study, supporting the hypothesis that monitoring endothelial function may be used to predict the risk of incident hypertension along with that of cardiovascular events.

## 4. Endothelial Function Evaluation

Given its systemic nature and potential reversibility in early stages, a number of laboratory and clinical methods have been proposed for endothelial function assessment and monitoring.

### 4.1. Laboratory Methods

In normal conditions, the endothelium has an anticoagulant, anti-inflammatory, and vasodilatory phenotype, which is reflected in the constitutive expression of NO, von Willebrand factor (vWF), plasminogen activator inhibitor-1 (PAI-1), and tissue factor (TF), as well as endothelium-derived adhesion molecules or chemokines, including intercellular adhesion molecule-1 (ICAM-1), vascular cell adhesion molecule-1 (VCAM-1), E-selectin, P-selectin, vascular endothelial-cadherin (VE-cadherin), and monocyte chemotactic protein-1 (MCP-1) [5]. The soluble forms of these endothelium-derived biomarkers can be measured in peripheral blood with different laboratory techniques.

More recently, the levels of some components of the glycocalyx (e.g., heparan sulfate, endocan, and syndecan-1) have been proposed as markers of endothelial function [78]. Finally, endothelial progenitor cells (EPCs) and circulating endothelial cells (CECs) have been used to test vascular repair capacity and the presence of endothelial injury [79].

### 4.2. Clinical Methods

Several clinical methods have been tested to assess endothelial function. If venous occlusion plethysmography (VOP) is substantially underutilized because of its invasiveness, laser doppler flowmetry (LDF) in cutaneous microcirculation has been increasingly employed in recent years [80]. Moreover, peripheral artery tonometry (PAT) has become a Food and Drug Administration (FDA)-approved test for an automated assessment of microvascular endothelial function [81]. Overall, although validated and highly reproducible, these methods may deal with the disadvantages of invasiveness and/or high cost, which may limit their use in routine clinical practice and sometimes in research [82].

**Figure 2:**
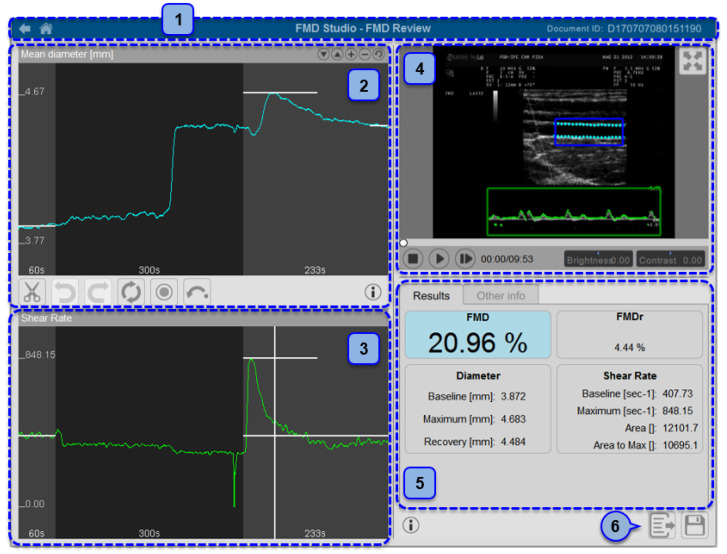
Automatic measurement of flow-mediated dilation (FMD) using a Food and Drug Administration (FDA)-cleared software. 1: Document identifier; 2: mean diameter; 3: shear rate; 4: video window; 5: results and info panel; 6: data exportation. Reproduced with permission from Quipu SRL, Pisa, Italy.

About 20 years ago, the guidelines for FMD assessment in conduit arteries were first reported by Corretti et al. [83]. Since then, the procedure has been increasingly used in clinical research, given its noninvasiveness and cost-effectiveness [5]. In brief, FMD is the percentage change in brachial artery diameter as a response to the shear stress induced by a pneumatic cuff placed on the forearm inflated to a suprasystolic pressure for 5 min. After cuff deflation, the increased blood flow enhances the shear stress on endothelium, which stimulates NO synthesis and, therefore, vasodilatation [83]. The fact that FMD has been widely recognized as a reliable surrogate marker of cardiovascular risk and an independent predictor of cardiovascular events [16], together with the recent identification of age- and sex-specific reference values [84], makes this method as one of the most used in clinical research studies. More recently, the use of a dedicated software for real-time edge detection, shear-rate monitoring, and wall tracking has proven to further increase reproducibility of FMD assessment [85] (Figure 2).

## 5. Therapeutic Targets for Endothelial Dysfunction in Hypertension

Whether endothelial and microvascular dysfunction may be causes or consequences of hypertension is still a matter of discussion [68]. However, the strict inter-relationship between the two conditions suggests that improving endothelial function may represent an attractive therapeutic target in the near future [86].

To date, renin–angiotensin system (RAS) inhibitors and statins are the main classes of drugs that have proven real effectiveness in reducing endothelial dysfunction in hypertension, thus restoring a vasodilatory and anticoagulant phenotype [87] while fighting microvascular dysfunction [88]. A number of other pharmacological and exercise-based strategies may positively impact endothelial homeostasis, microvascular function, and blood pressure. However, it is important to highlight that no routinely applied therapeutic approach can be considered specific to the endothelium, nor does any guideline currently recommend specific treatment in the presence of isolated endothelial dysfunction. Moreover, most of the novel promising candidates are still far from being translated into clinical practice [86]. Therefore, the impact on endothelium may be rather considered a welcome pleiotropic effect of many cardiovascular drugs and antihypertensive agents, including RAS inhibitors, calcium-channel blockers (CCBs), and β-blockers.

Here, we examined the main pharmacological, nutraceutical, and exercise-based approaches that a patient typically undergoes due to hypertension and cardiovascular comorbidities, focusing on the impact that these strategies may have on endothelial function as well.

### 5.1. ACE Inhibitors and Angiotensin II Receptor Blockers

ACE inhibitors and angiotensin II receptor blockers (ARBs) are key drugs in the treatment of hypertension, particularly in patients with multiple cardiovascular risk factors, heart failure, diabetes mellitus, and kidney impairment [89]. Therefore, given their capacity to reduce cardiovascular morbidity and mortality with an overall good tolerability [90,91], RAS inhibitors are widely prescribed as first-line drugs in essential hypertension [89] and coronary microvascular disease [88].

By reducing the synthesis of Ang II or blocking its AT1 receptor, these compounds are able to increase NO bioavailability, thus improving endothelial function [92] while also reducing the thrombotic risk due to their capacity to reduce TF and PAI-1 expression [93]. Overall, the lower stimulation of AT1 receptor results in a number of counter-regulatory actions on Ang II, also reducing inflammation and oxidative stress [13,48]. RAS inhibitors have been shown to increase the stability of eNOS mRNA while improving eNOS phosphorylation, reducing its uncoupling, reducing NOX expression, and increasing vascular levels of BH4 [94]. These drugs also lead to bradykinin accumulation in proximity of endothelial bradykinin receptors 1 and 2, which indirectly results in enhanced NO production [95]. Some studies also reported that, due to their antioxidant properties, RAS inhibitors have also an indirect effect on dimethylarginine dimethylaminohydrolase (DDAH), thus inducing the catabolism of asymmetric dimethylarginine (ADMA), which is an endogenous competitive inhibitor of eNOS [94].

Accordingly, using different outcome measures, including FMD, numerous clinical studies have demonstrated the ability of ACE inhibitors and ARBs to improve endothelial function [96,97], even in patients with concomitant coronary artery disease [98] and either type of diabetes mellitus [99,100].

Recently, the use of RAS inhibitors in COVID-19 has been put into question, given their capacity to upregulate ACE2 expression, thus hypothetically increasing the risk of infection. The evidence from randomized controlled trials of no difference in the risk of death between COVID-19 patients who use and those who do not use RAS inhibitors may be in line with the key pathogenic role of endothelial dysfunction in COVID-19 and with the capacity of these drugs to restore endothelial cells’ homeostasis.

### 5.2. Calcium-Channel Blockers

CCBs are another category of first-line drugs for arterial hypertension. The protective effect of CCBs on cardiovascular risk in hypertensive patients has been established [101], and the beneficial effect on endothelial function has also been documented [102], particularly in the coronary microvasculature [103]. The protective mechanism of CCBs on endothelial integrity is still unclear but it has been demonstrated that CCBs are able to counteract ROS-induced endothelial cell death due to lipid peroxidation [104]. In fact, although they may vary in their chemical structure and antihypertensive effect, dihydropyridine CCBs contain aromatic rings that stabilize oxygen radicals and possess a hydrogen-donating reaction, which may also account for their antioxidant activity [104]. A study from Napoli et al. [105] in hypertensive patients documented that CCBs reduced low-density lipoprotein (LDL) oxidation and formation of oxidation-specific epitopes, thus resulting in exalted antioxidant activity. CCBs also reduce calcium inflow in the voltage-dependent channels of subendothelial vascular smooth muscle cells, thereby resulting in vasodilation of large conduit and resistance arteries [106]. Moreover, dihydropyridine CCBs are able to inhibit the effects of endothelin-1 in the vascular smooth muscle, thus facilitating the vasodilatory activity of NO [106]. Another mechanism by which CCBs have shown a beneficial effect on vascular endothelium is their capacity to reduce tissue plasminogen activator (t-PA) activity [107].

### 5.3. β-Blockers

Similar to other antihypertensive drugs, β-blockers also exhibit a cardioprotective effect [108], particularly in patients with high resting heart rate or increased sympathetic tone and in those with coronary microvascular disease [109]. Using FMD as an outcome measure, nebivolol has shown a positive impact on endothelial function [110] and superiority as compared to atenolol, a selective β1-receptor blocker without vasodilatory properties [111]. β-blockers are able to inhibit fibrinogen, homocysteine, and PAI-1 while increasing NO levels via stimulation of eNOS [112]. Furthermore, some agents (i.e., carvedilol) may add an antioxidant effect, given their scavenging activity on ROS [110,113].

### 5.4. Other Cardiovascular Therapies

SAH is a traditional cardiovascular risk factor, which is frequently associated to a number of additional risk factors in the context of a dysmetabolic phenotype [114]. Therefore, given the presence of comorbidities (e.g., obesity, diabetes mellitus, dyslipidemia), the use of antihypertensive drugs is often associated to the use of hypoglycemic, hypolipidemic, and antithrombotic agents [114].

Statins may represent another promising approach targeting endothelial function, given a multitude of potential mechanisms, including the activation of eNOS via phosphatidylinositol-3 (PI3)-kinase/Akt pathway, the inhibition of nuclear factor-κB (NF-κB) and other inflammatory pathways, and the reduction in TF expression with subsequent anticoagulant effect [87]. In particular, it has been shown that statins are able to reduce the mRNA levels of AT1 receptor, thus reducing its expression [94]. Moreover, by improving vascular BH4 bioavailability, statins are able to improve eNOS coupling, thus resulting in increased NO bioavailability [115]. Another potential mechanism, in common with other hypolipidemic agents, may be the reduction in LDL cholesterol, thus contrasting the LDL-induced endothelial dysfunction and oxidative stress [116,117]. Therefore, different statins coupled with hypolipidemic diet have been tested in experimental murine and clinical models, documenting positive effects on endothelial function [118]. This is in line with the meta-analytical evidence that statin administration is able to improve FMD [119] while lowering blood concentrations of P-selectin, E-selectin, and ADMA [120,121]. 

Similarly, hypoglycemic agents targeting peroxisome proliferator-activated receptor-γ (PPAR-γ) have been shown to restore endothelial function in subjects with early phases of insulin resistance through prompting PI3-kinase/Akt/eNOS pathway and increasing NO production [122]. Thiazolidinediones exhibited the capacity to reduce NOX expression in animal models, thus lowering the production of ROS. Moreover, by reducing the expression of VCAM-1 and ICAM-1, they have been shown to limit the chemotaxis of macrophages and monocytes to the endothelium [94]. Given the above mechanisms, randomized clinical studies demonstrated that thiazolidinediones administration is able to significantly improve FMD in both diabetic and nondiabetic patients [123]. Similar beneficial effects on endothelial function have been documented with metformin and sodium-glucose cotransporter-2 (SGLT2) inhibitors [124,125], likely due to similar mechanisms [122].

Considering that platelets play a key role in atherogenesis, antiplatelets with different mechanisms of action are widely used for primary and secondary prevention of cardiac, cerebral, and peripheral ischemic complications. Although the possibility that their effectiveness may also depend on a beneficial effect on endothelium is still debated [126], cilostazol administration has already shown to improve FMD in conduit arteries [127] while suppressing Ang II-induced apoptosis in endothelial cells [128] and mobilizing EPCs [129]. Animal models demonstrated that the adenosine monophosphate-activated protein kinase (AMPK) activation may contribute to the beneficial effects of cilostazol on endothelial function [130].

### 5.5. Antioxidants and Nutraceutical Strategies

Given the inter-relationship between endothelial dysfunction and oxidative stress, antioxidant therapies may also be useful in restoring a normal endothelial function [131]. In this regard, glutathione and its precursor, namely N-acetyl cysteine (NAC), are potent antioxidants involved in the removal of H_2_O_2_ and other ROS [132]. Furthermore, NAC has anticoagulant properties and inhibits ACE2, thus reducing the deleterious effects of Ang II [133]. Accordingly, although traditionally used for its mucolytic properties, NAC may also have antihypertensive effect [134]. While NAC has been shown to inhibit atherosclerosis and improve endothelial function in animal models [135], the evidence in clinical trials is scarce and somehow conflicting [136,137].

A number of nutraceutical strategies, including vitamin supplementation, have shown beneficial effects on oxidative stress and endothelial dysfunction [138], while potentially reducing blood pressure [139]. In this regard, there is recent meta-analytical evidence of hypertensive patients having relatively low levels of vitamin C [140], with its supplementation significantly reducing blood pressure in SAH [141]. Vitamin C and vitamin E are free radical scavengers and are able to reduce lipid peroxidation, thus improving eNOS coupling and stabilizing BH4 [94]. However, contrasting results have been reported in large epidemiological studies regarding the impact that oral supplementation of vitamins may have on vascular health [94], considering that it may even reduce the lipid-lowering properties of statins and their beneficial effect on endothelial function [142]. Therefore, pending further high-quality evidence, the fact that vitamin supplementation may not have significant effects on blood pressure [143] is another reason not to impose the routine and indiscriminate use of vitamin supplements to improve vascular health and reduce cardiovascular risk.

Conflicting results have also been reported for the NO precursor L-arginine, which is able to antagonize the effect of ADMA on eNOS function. The fact that its favorable impact on endothelial function depends on the basal levels of ADMA may account for the lack of effect in healthy subjects [144]. However, while the positive impact of L-arginine supplementation on endothelial homeostasis seems to depend on the baseline status, it may conversely have a dose-dependent effect on both systolic and diastolic blood pressure regardless of the baseline blood pressure category (normotensive or hypertensive) [145].

Most of the future perspectives on the treatment of endothelial dysfunction in hypertension and cardiovascular disease relate to the possibility of targeting oxidative stress through epigenetic approaches (e.g., regulation of microRNAs levels, histone acetylation/methylation, DNA methylation), the implementation of new pharmacological strategies targeting the oxidatively impaired eNOS or soluble guanylate cyclase, or the delivery of antioxidants directly to the endothelium through specific ligands or using vectors (e.g., liposomes) [86]. Although promising results have been obtained in vitro or in animal models, these strategies are still far from being translated into clinical practice.

### 5.6. Exercise and Rehabilitation

Rehabilitation has already proven its effectiveness in improving functional capacity, exercise performance, symptoms, and health-related quality of life in different clinical settings [146,147,148], while reducing the risk of exacerbations in chronic obstructive pulmonary disease (COPD) [149,150] and cardiovascular mortality in coronary artery disease [151,152]. Moreover, exercise-based approaches have been proposed also as promising interventions to modify the course of cerebral small vessel diseases and improve microvascular responsiveness [153,154]. Although not representing the primary outcome of multidisciplinary rehabilitation, the positive impact on blood pressure has been reported as a welcome pleiotropic effect of exercise-based approaches [155].

In the 1980s, the beneficial effect of exercise on endothelial function was first documented [156], being later confirmed in several reports focusing on specific clinical settings, including cardiovascular conditions [151,152]. Cardiac rehabilitation has a Class I recommendation in most guidelines [157], and the impact on endothelial function has been mainly tested in acute myocardial infarction, stable coronary artery disease, and heart failure [158,159,160,161]. Although with variable results, these studies agree on the beneficial effect of cardiac rehabilitation on endothelial function, particularly when it is significantly impaired. Similar findings have been recently reported when testing FMD in COPD patients undergoing pulmonary rehabilitation [162]. Some mechanisms have been proposed in this regard, including reduced uncoupling and increased eNOS phosphorylation, upregulation of superoxide dismutase, NOX downregulation, and EPCs mobilization [163]. It is reasonable to assume that the positive impact of exercise-based approaches on endothelial function may somehow account for the positive impact that these strategies may have also on blood pressure control [155]. This should be taken into consideration, given the evidence that hypertension is a frequent comorbid condition of the diseases that usually require rehabilitation programs [164,165]. More recently, the positive impact of in-hospital rehabilitation on both endothelial function and arterial blood pressure has been demonstrated also in the new coronavirus disease 2019 (COVID-19), with a potential reduction in the residual cardiovascular risk of COVID-19 survivors [166].

## 6. Conclusions

The relationship between systemic arterial hypertension and endothelial dysfunction comprises a bidirectional connection, which amplifies the magnitude of each factor. Endothelial cells, through canonical mediators and other paracrin systems, influence the pathogenesis of systemic hypertension. Similarly, systemic hypertension promotes endothelial dysfunction and contributes to the prothrombotic and proinflammatory status, increasing the cardiovascular risk. Endothelial dysfunction assessment among hypertensive patients may offer a new paradigm to define a specific cardiovascular phenotype with higher risk and should be carefully taken into consideration in clinical practice. Current agents, targeting systemic hypertension and metabolic disorders, may improve endothelial dysfunction by both favoring NO availability and restoring antioxidant properties. The fine modulation of these complex pathways is of primary importance based on the above reported data.

## Data Availability

No new data were analyzed in this study.
